# *SNAP-25* Single Nucleotide Polymorphisms, Brain Morphology and Intelligence in Children With Borderline Intellectual Functioning: A Mediation Analysis

**DOI:** 10.3389/fnins.2021.715048

**Published:** 2021-08-26

**Authors:** Valeria Blasi, Elisabetta Bolognesi, Cristian Ricci, Gisella Baglio, Milena Zanzottera, Maria Paola Canevini, Mauro Walder, Monia Cabinio, Michela Zanette, Francesca Baglio, Mario Clerici, Franca Rosa Guerini

**Affiliations:** ^1^IRCCS Fondazione Don Carlo Gnocchi ONLUS, Milan, Italy; ^2^Pediatric Epidemiology, Department of Pediatrics, Medical Faculty, Leipzig University, Leipzig, Germany; ^3^Epilepsy Center, ASST S. Paolo and S. Carlo Hospital, Milan, Italy; ^4^Department of Health Sciences, University of Milan, Milan, Italy; ^5^Child Neuropsychiatry Unit – ASST S. Paolo and S. Carlo Hospital, Milan, Italy; ^6^Department of Pathophysiology and Transplantation, University of Milan, Milan, Italy

**Keywords:** genetics, brain imaging, learning disabilities, neural plasticity, rehabilitation, child psychiatry

## Abstract

Borderline intellectual functioning (BIF) is a multifactorial condition in which both genetic and environmental factors are likely to contribute to the clinical outcome. Abnormal cortical development and lower IQ scores were shown to be correlated in BIF children, but the genetic components of this condition and their possible connection with intelligence and brain morphology have never been investigated in BIF. The synaptosomal-associated protein of 25 kD (SNAP-25) is involved in synaptic plasticity, neural maturation, and neurotransmission, affecting intellectual functioning. We investigated *SNAP-25* polymorphisms in BIF and correlated such polymorphisms with intelligence and cortical thickness, using socioeconomic status and environmental stress as covariates as a good proxy of the variables that determine intellectual abilities. Thirty-three children with a diagnosis of BIF were enrolled in the study. *SNAP-25* polymorphisms rs363050, rs363039, rs363043, rs3746544, and rs1051312 were analyzed by genotyping; cortical thickness was studied by MRI; intelligence was measured using the WISC-III/IV subscales; environmental stressors playing a role in neuropsychiatric development were considered as covariate factors. Results showed that BIF children carrying the rs363043(T) minor allele represented by (CT + TT) genotypes were characterized by lower performance Perceptual Reasoning Index and lower full-scale IQ scores (*p* = 0.04) compared to those carrying the (CC) genotype. This association was correlated with a reduced thickness of the left inferior parietal cortex (direct effect = 0.44) and of the left supramarginal gyrus (direct effect = 0.56). These results suggest a link between *SNAP-25* polymorphism and intelligence with the mediation role of brain morphological features in children with BIF.

## Introduction

Borderline intellectual functioning (BIF) is a condition characterized by a mental ability at the border between normal intellectual functioning and intellectual disability, with an Intellectual Quotient (IQ) within 1 and 2 standard deviations below the mean of the normal curve of the distribution of intelligence that impacts on adaptive abilities (Salvador-Carulla et al., 2013; Peltopuro et al., 2014). In primary school age, children with BIF are burdened with difficulties in school achievements due to learning difficulties in more than one executive functions domain, such as attention, concentration, planning, and inhibition of impulsive responses, as well as memory and motor skill limitations (Alloway, 2010; Vuijk et al., 2010; Salvador-Carulla et al., 2013; Peltopuro et al., 2014; Contena and Taddei, 2017). Furthermore, limitations in social skills, emotional competencies, and behavioral problems affect the social participation of these children (Baglio et al., 2016; Predescu et al., 2020). Children with BIF are thus at high risk of school failure and dropout (Fernell and Ek, 2010), and are more likely to develop psychiatric problems in adulthood (Douma et al., 2007; Emerson et al., 2010; Gigi et al., 2014; Hassiotis, 2015; Hassiotis et al., 2019). Potential risk factors for BIF include low weight at birth, low socioeconomic status, maltreatment, and high levels of maternal stress. However, the negative social condition does not explain all the BIF cases and their development across the life span.

Intelligence is one of the most heritable behavioral traits (Deary et al., 2009). Intelligence is nevertheless also a malleable entity under the influence of environmental conditions (Sauce and Matzel, 2018). As a logical consequence of this, intellectual disability, as well as the development of psychiatric disorders were suggested to be the result of an interaction between social environment and genetic background (Rizzi et al., 2012). Finally, a multifactorial and multigenic set may be responsible for BIF development (Ropers, 2008).

An important role in intelligence is likely played by the synaptosomal-associated protein of 25 kD (*SNAP-25*) gene, which is located on chromosome 20p12-p11.2, an area of previous suggestive linkage to intelligence (Posthuma et al., 2005). SNAP-25 protein takes part in the regulation of calcium-dependent synaptic vesicles exocytosis, ensuring the efficient release of neurotransmitters and the propagation of action potentials. The key role of SNAP-25 is to initiate exocytosis through the formation of a SNARE complex (Südhof, 2015). The SNARE complex is therefore involved in the processes of learning, locomotion, memory formation, and ultimately the normal functioning of the brain as a whole. *SNAP-25* single nucleotide polymorphisms (SNPs) were associated with variation of performance IQ in non-clinical, population based samples (Gosso et al., 2006, 2008). Interestingly, polymorphisms in the *SNAP-25* gene, as well as an altered expression of the SNAP-25 protein, are also associated with abnormal behavioral phenotype both in humans (Thompson et al., 2003; Guerini et al., 2011; Braida et al., 2015; Liu et al., 2017) and in animal models (Bruno et al., 2006; Gunn et al., 2011). Finally, evidence derived from multiple organisms suggested that SNAP-25 is involved in the process of axonal growth and synaptic plasticity (Martinez-Arca et al., 2001). Therefore, any variation of SNAP-25 protein expression may interfere with neural maturation and neurotransmission, affecting intellectual functioning.

Neural plasticity during development was investigated with neuroimaging techniques that evaluated longitudinal changes in cortical thickness, a parameter influenced by genetics, which modulates intelligence (Brans et al., 2010). Notably, a number of results show how differences in a distributed network that include frontal and parietal cortices predict individual profiles in intelligence (Jung and Haier, 2007). Earlier data from our group, in particular, showed the presence of significant differences in cortical volume in areas belonging to this network in children with BIF and the relationship of this difference with intelligence (Baglio et al., 2014).

The aim of our work was to examine the complex relation between *SNAP-25* and cortical thickness in determining intelligence. To accomplish this aim, we selected four SNPs located in the intron region which were proved to be involved in both typical (Gosso et al., 2006, 2008) and atypical development (Barr et al., 2000; Guerini et al., 2011; Braida et al., 2015). We first identified the presence of possible correlations between the *SNAP-25* rs363050, rs363039, rs363043, rs3746544, and rs1051312 genetic polymorphisms with brain area morphology, and IQ scale in 33 children with BIF. Next, we conducted a mediation analysis in which genetic polymorphisms, brain area morphology and IQ scale were modeled in a comprehensive fashion. An association of the *SNAP-25* rs363043 polymorphism with PRI as well as with IQ scores was reported to be mediated by brain cortical thickness in the inferior parietal lobule.

## Materials and Methods

### Patients Enrolled in the Study

Children were recruited from the Child and Adolescent Neuropsychiatry Unit of IRCCS Don Carlo Gnocchi Foundation and the ASST S. Paolo and S. Carlo Hospital; both in Milan, Italy. The sample included thirty-three children (6–11 years old) with BIF, i.e., a Full-Scale Intelligence Quotient (FSIQ) score in the borderline range, and clinical criteria, that attend primary mainstream school. The clinical evaluation consisted of a detailed medical history and social skills of the child and of his/her family and clinical observations reports.

Excluded criteria were: (1) ADHD, autism spectrum disorder, or other major neuropsychiatric disorders; (2) epilepsy, traumatic brain injury, brain malformation, infectious disease and other neurological conditions involving the central nervous system, and perinatal complications such as prematurity or other adverse events; (3) genetic syndromes such as Down syndrome or Fragile X syndrome and (4) systemic diseases such as diabetes or dysimmune disorders; and (5) current or past substance abuse (psychoactive drugs, psycho stimulants, neuroleptics, antidepressants, benzodiazepines, and antiepileptic drugs).

Informed consent was obtained from all parents/legal guardians prior to inclusion in the study. The study was conducted according to the guidelines of the Declaration of Helsinki and was approved by the institutional review board of the Don Carlo Gnocchi ONLUS Foundation, Milan (Protocol nr: 06_18-05-2016).

Socioeconomic status was assessed by the SES questionnaire, an integrated measure of parent’s education grade and occupation, widely used in research to identify the child/family social standing. The socio-cultural levels are low (range 8–19), middle-low (range 20–29), middle (range 30–39), middle-high (range 40–54) and high (range 55–66) (Hollingshead, 2011). Environmental stress was defined according to the ESCL, a list of V-codes from DSM-5, and Z-codes from ICD-10, to detect relational, neglect, physical, sexual and/or psychological abuse, educational and occupational, housing and economic, social exclusion or rejection problems, plus the presence of the following three conditions: social services intervention, major psychiatric diagnosis and/or substance abuse within the family members. The scoring is binary with a 0 (absence) or 1 (presence) attribution to each item, with a total score ranging from 0 to 24 (Blasi et al., 2020).

### Neuropsychological Evaluation

The neuropsychological evaluation included: (1) the WISC –III (Orsini and Picone, 2006), with the exception of nine children evaluated with the WISC-IV (Orsini et al., 2012) that assess the global intellectual functioning and the cognitive profile; (2) the Socioeconomic Status (SES) (Hollingshead, 2011), to evaluate the family education level and financial well-being; (3) the Environmental Stress Check List (ESCL), a tool to detect all possible sources of environmental stress (Blasi et al., 2020) to which the children were exposed to.

The WISC-III provides three principal scores: the FSIQ, the Verbal IQ (VIQ), and the Performance (PIQ); in addition, to better describe the cognitive profile, it is possible to calculate four indices: the Verbal Comprehension Index (VCI), the Perceptual Organization Index (POI), the Freedom from Distractibility Index (FDI) and the Processing Speed Index (PSI). The WISC–IV provides an FSIQ and a four-index framework similar to that of the WISC III: the VCI, the Perceptual Reasoning Index (PRI), the Working Memory Index (WMI), and the PSI. The increased emphasis on fluid reasoning abilities and on working memory, with the introduction of new subtests, has resulted in the renaming of the POI as the Perceptual Reasoning Index (PRI) and the FDI as the Working Memory Index (WMI) respectively. Moreover, a high correlation between FSIQ, VCI, and PRI of both versions is demonstrated (FSIQ-FSIQ = 0.89; VCI-VCI = 0.88; POI-PRI = 0.72) (Flanagan and Kaufman, 2012). Finally, all indices are expressed in the standard score (Mean = 100; SD = 15). For the present study, we will refer to both POI and PRI indices with the acronym PRI and to both FDI and WMI as WMI. All the data were age-corrected when measured.

### MRI Acquisition

All subjects underwent a magnetic resonance imaging (MRI) evaluation. MRI was performed on a 1.5 T Siemens Magnetom Avanto (Erlangen, Germany) scanner equipped with a 12-channels head coil. The acquisition included: (1) a 3D T1-weighted Magnetization Prepared Rapid Gradient-Echo (MPRAGE) image, (repetition time (TR)/echo time (TE) = 1900/3.37 ms, Filed of View (FoV) = 192 × 256 mm^2^, voxel size = 1 mm isotropic, 176 axial slices); (2) two conventional anatomical sequences (axial PD/T2 and coronal FLAIR) to exclude gross brain abnormalities.

### MRI Data Analysis

The 3D-T1 images were segmented and parcellated using FreeSurfer version 5.3^1^ into 68 cortical areas (34 for each hemisphere) according to the Desikan atlas (Desikan et al., 2006). Furthermore, the FreeSurfer automatic labeling process was used to extract seven subcortical regions per hemisphere (thalamus, caudate, putamen, pallidum, and nucleus accumbens, amygdala, and hippocampus) and the brain stem for a total of 82 parcels. The quality of recon-all parcelation was assessed in each subject according to ENIGMA guidelines^2^ for cortical and subcortical areas.

Mean thickness was then computed for each cortical area, and mean volume for each subcortical region.

### Genetic Analyses

Single nucleotide polymorphisms typing: Three *SNAP-25* SNPs: rs363050, rs363039, and rs363043 located within intron 1, in a region of about 13.8 kb, known to affect transcription factor binding sites (Gosso et al., 2008) as well as two *SNAP-25* SNPs: rs3746544 and rs1051312 located in the 3′ untranslated region predicted as a binding site of miRNAs (endogenous non-coding RNA regulators of gene activity at the post-transcriptional level) (Ambros, 2004; Bartel, 2004) were investigated; these SNPs have previously been associated with ADHD (Barr et al., 2000). Genomic DNA was isolated from peripheral blood mononuclear cells by phenol-chloroform extraction. SNPs were typed using the Taqman SNP Genotyping Assays (Applied Biosystems by Life Technologies, Foster City, CA, United States) on an ABI PRISM 7000 Sequence Detection System. For rs363039, rs363043, rs363050, and rs3746544, respectively, the C_327976_10, C_2488346_10, C_329097_10 and C_27494002_10 Human Pre-Designed Assays (Applied Biosystems by Life Technologies) were used. The restriction enzyme polymorphism rs1051312 was genotyped by *Dde*I digestion as previously described (Barr et al., 2000).

### Statistical Analysis

Age, socioeconomic status, environmental stress index, the total score, and the subscales of the Wechsler Intelligence Scale for Children were described by median and 5th to 95th centile range. *SNAP-25* polymorphisms were described by frequencies (Table 1).

**TABLE 1:** Median, 5th–95th centiles of age, socioeconomic status, environmental stress and psychometric scores.

	Overall, *N* = 33	Boys, *N* = 19 (57.6%)	Girls, *N* = 14 (42.4%)
Age (years)	9.0 (6.0,10.0)	8.0 (6.0,10.1)	9.0 (6.0,10.0)
SES	22.0 (14.0,45.2)	22.0 (15.6,51.0)	22.0 (14.0,32.7)
ESCL	4.0 (0.6,8.0)	4.0 (1.0,8.2)	2.5 (0.0,8.0)
**WISC-III-IV**			
FSIQ	78.0 (62.8,85.4)	80.0 (69.1,86.2)	76.0 (62.3,85.0)
VCI	80.0 (57.0,94.8)	84.0 (68.2,94.4)	78.5 (57.3,89.5)
PRI	84.0 (77.2,104.2)	85.0 (77.8,103.8)	83.0 (74.9,98.9)
WMI	75.0 (63.0,92.2)	75.0 (61.8,94.6)	76.5 (63.0,91.0)
PSI	77.0 (60.8,95.2)	74.0 (58.7,94.6)	77.3 (64.0,95.0)
***SNAP-25***			
rs363039	GG (11), GA + AA (22)	GG (7), GA + AA (12)	GG (4), GA + AA (12)
rs363043	CC (16), CT + TT (17)	CC (8), CT + TT (11)	CC (8), CT + TT (6)
rs363050	AA (14), AG + GG (19)	AA (8), AG + GG (11)	AA (6), AG + GG (10)
rs3746544	TT (11), TG + GG (22)	TT (6), TG + GG (13)	TT (5), TG + GG (9)
rs1051312	TT (25), TC + CC (8)	TT (16), TC + CC (3)	TT (9), TC + CC (5)

*Frequencies were reported for *SNAP-25* polymorphisms.*

*SES, socioeconomic status; ESCL, environmental stress check list score; WISC-III-IV, Wechsler Intelligence Scale for Children; FSIQ, Full-Scale Intelligence Quotient; VCI, Verbal Comprehension Index; PRI, Perceptual Reasoning Index; WMI, Working Memory index; PSI, Processing Speed Index.*

Exact Hardy Weinberg analysis was applied to verify if *SNAP-25* SNPs genotype distribution among children with BIF were in Equilibrium (HWE).

*SNAP-25* Linkage disequilibrium was calculated by SHEsis (Shi and He, 2005) by adding data from 615 healthy control from a previous study (Guerini et al., 2014). Haplotype correlation with IQ profile was calculated by regression analysis, adjusting by gender, SES, and ESCL, using PLINK software (Purcell et al., 2007).

A causal network approach was applied to investigate the association between *SNAP-25* SNPs, MRI morphological data, and WISC-III-IV subscales (FSIQ, VCI, PRI, WMI, PSI) (Agler and De Boeck, 2017). Specifically, we considered a network in which *SNAP-25* SNPs may act on psychometric scores through a direct relation and by an indirect pathway in which morphological data, measured by MRI, may act as a mediator. Firstly, to reduce skewness, all outcome data were standardized and transformed in normal ranks using Blom’s transformation (Gumbel, 1959). Afterward, models bearing the direct association (*SNAP-25* to WISC III-IV subscale) and two indirect associations (*SNAP-25* to brain morphology and brain morphology to WISC III-IV subscale), were applied to investigate the above causal pathway. Those models were based on a Kernel not parametric regression adjusted for sex, socioeconomic status, and Environmental Stress Check List score (ESCL). Regression coefficients were estimated and tested for significance by a procedure based on 5,000 bootstrap replications. To take into account numerous comparisons and to manage the related false discovery rate, the threshold *P*-value to detect significant associations was set according to the Benjamini–Hochberg procedure (Chen et al., 2017). Briefly, this method was chosen, instead of more conservative approaches, because of the small sample size and the need to reduce the false-negative rate (reduce the type-II error). Heat maps of statistically significant results were the graphical tool chosen to represent multiple associations between *SNAP-25* SNPs, MRI morphological data, and psychometric scores.

Afterward, according to the above explorative analyses, a mediation analysis was used to investigate the role of brain morphology in the association between *SNAP-25* SNPs and psychometric scores. In the mediation analyses, the direct and indirect effects were reported as standardized and rescaled regression coefficients. The total effect of *SNAP-25* SNPs on psychometric scores was computed as a + b × c and as b × c as the total and indirect effects respectively. Here the term “a” was the slope of the direct effect, while the terms “b” and “c” were the slopes of the two sides of the indirect effects, the association between single nucleotide polymorphism with MRI data, and the association between MRI data and the psychometric scores, respectively. Standardized coefficients were reported for all the associations. All statistical tests were two-tailed and the type-I error rate was set according to the Benjamini–Hochberg procedure for the single Kernel non-parametric models while an ordinary type-I error rate of 5% (α = 0.05) was considered for the mediation analyses. The NP and the LAVAAN packages of the R software vers. 3.6. were used to conduct the kernel non-parametric regressions and the mediation analysis, respectively.

Given the small sample size, *a priori* and *a posteriori* power calculations were performed. In particular, the statistical power of Kernel non-parametric regression was investigated using the MultNonParam package of the R software vers. 3.6 while the statistical power of the mediation analysis was investigated using the pwr package of the R software vers. 3.6. According to this evaluation and considering the current sample size and type-I error rate used, medium to large standardized effect size in the range of 0.3–0.5, could be detected with a type-II error rate below 20% (Statistical power above 80%) for those two methods. According to statistical power, the current study could be defined as explorative.

## Results

### *SNAP-25* Polymorphisms Association With Neuropsychological Scores

The sample was composed of 19 boys and 14 girls with a median age of 9 years (5th to 95th centile 6–10 years old). Age, socioeconomic status, IQ evaluation scores, and *SNAP-25* polymorphisms were similar in boys and girls; the ESCL score, on the other hand, was lower in girls compared to boys, although not significantly (*p* = 0.3964). All WISC-III-IV scores had a median lower than 85, below 1 standard deviation with respect to the normal population (Table 1).

**FIGURE 1:**
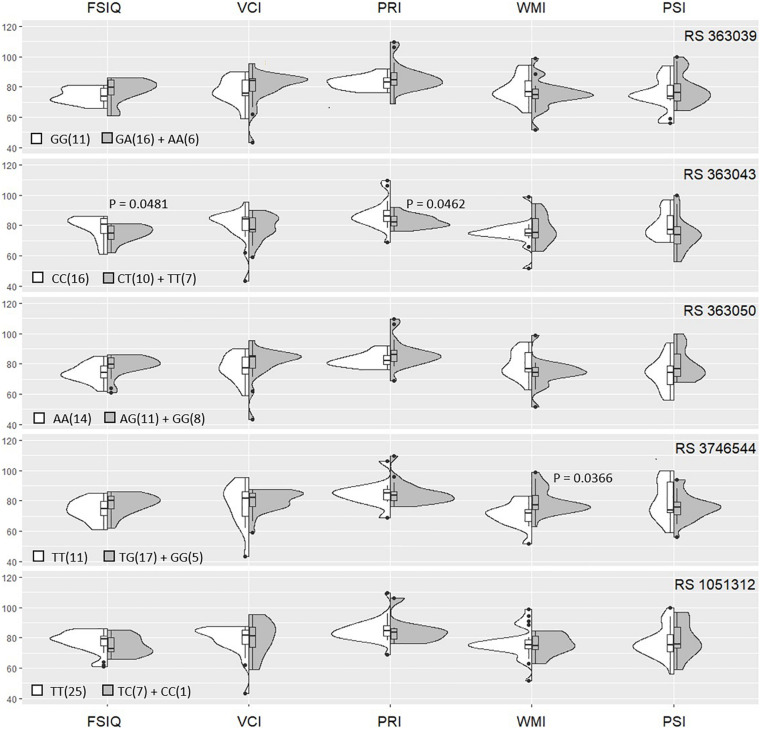
Violin plots of Kernel’s smoothed distributions of WISC-III/IV scores by *SNAP-25* genotypes. The box plots present the distribution of each IQ score (median and interquartile range) relatively to the genotype distribution. The “violin” area indicate the percentage of children with BIF for each allele (in gray the minor allele and white the major allele).

Initial results showed that *SNAP-25* rs363039, rs363043, rs363050, rs3746544, and rs1051312 SNPs genotype distribution was in HWE (*p* = 0.96; *p* = 0.05; *p* = 0.07; *p* = 0.71; *p* = 0.57 respectively) (Figure 1). The *SNAP-25* genetic patterns of distribution in BIF children, clustered into carriers of the minor allele (i.e., heterozygous + homozygous for minor) and carriers of the homozygote major allele, were analyzed next in relationship with FSIQ, VCI, PRI, WMI, and PSI scores (Figure 1).

Analysis of the relation between *SNAP-25* polymorphisms and IQ scores showed the presence of a significant association between the rs363043 (T) minor allele represented by (CT + TT) genotypes and lower FSIQ and PRI scores (*p* = 0.04). Notably, additional results showed that the rs3746544 (TT) genotype was also significantly associated with reduced WMI scores (*p* = 0.04) (Figure 1).

### *SNAP-25* Haplotype Linkage Analyses

Linkage haplotype analyses were used to evaluate the linkage disequilibrium between *SNAP-25* variants, as well as to verify the presence of an association between the different haplotypes and FSIQ, VCI, PRI, WMI, and PSI scores. Haplotype analysis evidenced, the presence of a linkage disequilibrium (LD) between: (1) rs363050 and rs363039 (*r*2 > 0.3); (2) rs363043 and rs363050 (*r*2 = 0.24); and (3) rs363043 and rs363039 (*r*2 = 0.17) and a linkage between rs3746544 and rs105312 polymorphisms (*r*2 = 0.17). Haplotype distributions were not significantly associated with IQ scores (data not shown).

### MRI Parameters Mediators Between *SNAP-25* Polymorphisms and Intelligence

The relations between *SNAP-25* polymorphisms and MRI morphological data, along with the relations between MRI morphological data from IQ evaluation were analyzed next. Results showed the presence of a number of associations between these features (Supplementary Figures 1, 2). In particular, this exploratory association identified the cortical thickness in the left inferior parietal cortex as a possible mediator between rs363043 and PRI scores. Moreover, the thickness of the left supramarginal gyrus was suggested to act as a possible mediator between rs363043 and FSIQ. Finally, when considering the relation between the PRI score with the left inferior parietal cortex a significant variance of 21% was observed, whereas the relation between FSIQ with the left supramarginal gyrus resulted in an explained variance of 24%.

**FIGURE 2:**
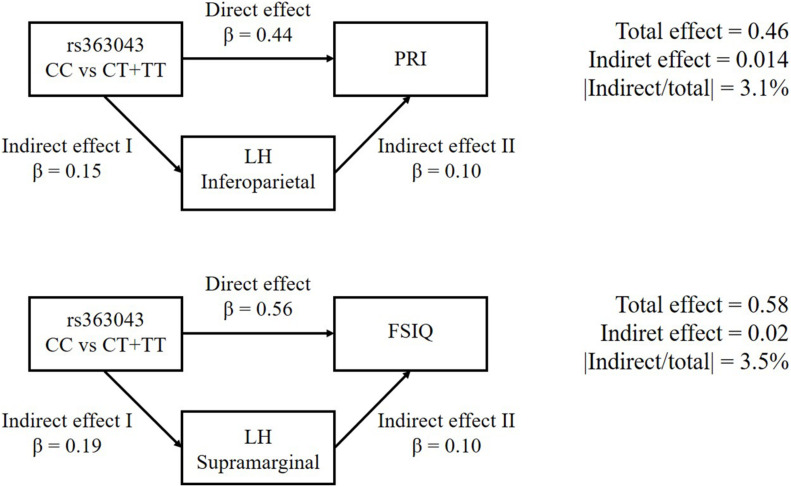
Causal mediation analyses of *SNAP-25* rs363043 on PRI and FSIQ scores of the WISC-III/IV. The Left inferoparietal and supramarginal cortex thickness as the mediators for PRI and FSIQ, respectively. The Left inferior parietal and supramarginal cortex thickness were observed to be the mediators for PRI and FSIQ, respectively. The upper part of the figure shows the association of the rs363043 polymorphism with PRI score (direct effect = 0.44; contribution of the left inferior parietal = 3.1%). The lower part of the figure shows the association of the rs363043 polymorphism with FSIQ score (direct effect = 0.56; contribution of the left Supramarginal gyrus = 3.5%).

According to our mediation analyses, the association of the rs363043 polymorphism influenced the PRI score with a direct effect of 0.44. This association was mediated by a cortical thickness of the left inferior parietal by 3.1%. This means that a lower score of PRI associated with rs363043 (CT + TT) genotypes is directly related to a reduced cortical thickness. Similarly, the association of the rs363043 polymorphism with the FSIQ score had a direct effect of 0.56 and a mediated indirect effect of the cortical thickness of the left supramarginal gyrus of 3.5%. This means that the lower FSIQ score associated with the rs363043 (CT + TT) genotypes is directly related to a reduced cortical thickness of the left supramarginal gyrus. Results of the mediation analyses are presented in Figure 2.

## Discussion

In the present study, we show that *SNAP-25* rs363043(T) minor allele represented by (CT + TT) genotypes are associated with lower PRI scores in children with BIF; such association was found to be mediated by the left inferior parietal cortex thickness: lower thickness mediates lower PRI scores. Notably, the same children carrying the rs363043 (CT) or (TT) genotypes showed a lower full-scale IQ score, and this association was mediated by the cortical thickness in the left supramarginal gyrus.

The *SNAP-25* SNP rs363043, along with other polymorphisms on the *SNAP-25* gene, has previously been associated with intelligence in a normal population of Dutch children, adolescents, and adults (Gosso et al., 2006, 2008). In that case, though, the rs363043 (T) allele was associated with higher Verbal and performance IQ. Differences in the populations that have been analyzed, as well as in environmental components, might explain these discrepant results. Specifically, in the previous work (Gosso et al., 2008) the authors studied a population with an average IQ while we analyzed a group of BIF children belonging to a low socioeconomic status; importantly, the effect of several environmental stressors was considered as well in our analyses.

The interplay between genetic and environmental factors is complex and most likely both these factors have an important impact on individual differences in IQ (Sauce and Matzel, 2018). In the attempt to consider both aspects, we designed a model that includes the socioeconomic status and environmental stress as covariates; we believe this approach to be a good proxy of the diverse variables that determine intellectual abilities.

The *SNAP-25* rs363043 polymorphism herein reported is a (non) coding variant within the intron 1 of the *SNAP-25* gene that was shown to be involved in the regulation of SNAP-25 protein expression (Gosso et al., 2006). SNAP-25 protein is differentially expressed in the brain and is primarily present in the cerebral cortex, cerebellum, hippocampus, and caudate^3^. Notably, chronic reduction of SNAP-25 expression was shown to affect behavior in animal models. Thus, the coloboma mouse model, characterized by halved SNAP-25 levels (Hess et al., 1992), displays a hyperactive phenotype (Hess et al., 1992), associated with abnormal thalamic spike-wave discharges (Hess et al., 1995; Zhang et al., 2004; Faraone et al., 2005; Russell, 2007). Similarly, juvenile SNAP-25 heterozygous mice display moderate hyperactivity, which disappears in the adult animals, as well as impaired associative learning and memory, which persist in adulthood (Corradini et al., 2014).

Multiple studies have shown that different *SNAP-25* SNPs are associated with related traits of autism (Guerini et al., 2011) and ADHD (Forero et al., 2009; Braida et al., 2015), as well as with working memory ability (Söderqvist et al., 2010; Gao et al., 2015), short-/long-term memory and visual attention (Golimbet et al., 2010), and intellectual disability (Rizzi et al., 2012), These observations can be explained by the fact that the markers studied here are located close to a locus linked with behavioral and cognitive functions. A genetic linkage disequilibrium effect, which would explain the involvement of different SNPs in the same *SNAP-25* genetic locus cannot be excluded either in our cohort of BIF children.

In the attempt to find an explanation for our results, we investigated if *SNAP-25* SNPs could influence differences in brain morphology, thus explaining the connection between genetics and IQ scores. Results showed the involvement of different areas in the left hemisphere. In particular, the left inferior parietal cortex and the left supramarginal gyrus were found to mediate between genetics and the PRI and the FSIQ respectively. The PRI is a measure of the non-verbal components of intelligence such as visuospatial and visuomotor abilities involved in the reasoning and solving of new problems, while FSIQ is a composite measure derived by all IQ scores relative to verbal and non-verbal abilities. Interestingly, we observed that the relationship between PRI and FSIQ and genetics was mediated by the inferior parietal cortex and the supramarginal gyrus, which are both parts of the inferior parietal lobule (IPL), a multimodal region, considered a hub for its great interconnectivity with several areas in the brain (Igelström and Graziano, 2017). Our results are in line with previous evidence showing how the IPL has a relevant role in multimodal information integration for higher-order cognitive functions such as abstraction and symbolization (Igelström and Graziano, 2017). Moreover, the IPL is part of the mirror neuron system (Rizzolatti and Craighero, 2004), which is involved in the visuomotor integration processing of gestures, relevant not only for action understanding but also for learning. In support of these data, previous results showed how multimodal rehabilitation interventions to improve the intellectual abilities of children with BIF were more effective than single domain treatment (Blasi et al., 2020). Notably, our results can be seen in the light of previous studies showing how the variation in the cortical thickness of a distributed network comprising the dorsolateral prefrontal cortex, the inferior and superior parietal lobule, the anterior cingulate, and regions within the temporal and occipital lobes, predicts individual differences in the g-factor of intelligence (Jung and Haier, 2007). Specifically, a positive correlation between intelligence and cortical thickness in the IPL has been demonstrated in both children and adults (Menary et al., 2013).

Results herein are in line with previous studies showing that individual differences in frontal and parietal cortical thickness are strongly influenced by genetic components (Posthuma et al., 2002). The interplay between genetic and environment is complex and most likely both these factors have an important impact on individual differences in IQ (Sauce and Matzel, 2018). In our study, all children belonged to high-risk environments, had a medium to low SES, and were undergoing the effect of environmental stressors, all factors indirectly associated with low thickness in the frontoparietal network (Rosen et al., 2018). Our data suggest that the genetic background interacts with environmental factors in shaping brain configuration, thus determining the outcome of BIF.

### Strength and Limitations

The current work has remarkable strengths. Firstly, we investigated determinants of intelligence in a sample of children with borderline intellectual functioning, a population of great clinical interest. Secondly, we adopted a rigorous research methodology resulting in reliable genetic polymorphisms, measures of intelligence, and brain morphology. Moreover, we used a comprehensive approach that included genetic, brain morphology, and intelligence outcomes as a whole, in an integrated analytical framework in which the issue of false discovery rate given by the limited sample size and large numbers of comparisons were taken into account using the appropriate statistical approaches. Finally, we would like to underline that this is the first study conducted in children with BIF that describes a multimodal association between *SNAP-25* polymorphisms, intelligence, and brain morphological features.

As usually is the case, though, this work also has limitations. Firstly, results were drawn from analyses performed in a limited number of children which could result in false-negative results. Moreover, the lack of a deeper analysis of the *SNAP-25* gene by next generation sequencing does not allow us to exclude that other polymorphisms could also be involved in shaping the intellectual functioning of children with BIF. Future studies evaluating the rs363043 polymorphism regulatory ability in larger cohorts of patients as well as an expression analysis of *SNAP-25* gene would be necessary.

Further, the observational nature of the study, the reduced sample size, and the skewness of the outcomes considered led to the use of non-parametric multivariate-adjusted models; this might have reduced the statistical power of statistical analyses. The use of Benjamini–Hochberg procedure to adjust for multiple comparisons and the use of bootstrap, nevertheless likely took care of this issue. Finally, without a control group, our study could be defined just as an explorative attempt. More rigorous observational studies, with larger sample size and possibly based on a matched case-control design, should be performed to validate our results.

## Data Availability Statement

The data presented in this study are available on request from the corresponding author. The data are not publicly available due to privacy concerns.

## Ethics Statement

The studies involving human participants were reviewed and approved by the Ethics Committees of the Don Gnocchi Foundation. Written informed consent to participate in this study was provided by the participants’ legal guardian/next of kin.

## Author Contributions

FG, VB, MaC, and EB: conceptualization. FG, VB, CR, and EB: methodology. EB, MilZ, GB, and MoC: formal analysis. FG, VB, FB, EB, CR, MoC, and GB: investigation. FG and VB: data curation, writing—original draft preparation, and project administration. MPC, MW, and MicZ: resources. FG, VB, CR, and MaC: writing—review and editing. FG, VB, MaC, and FB: funding acquisition. All authors have read and agreed to the published version of the manuscript.

## Conflict of Interest

The authors declare that the research was conducted in the absence of any commercial or financial relationships that could be construed as a potential conflict of interest.

## Publisher’s Note

All claims expressed in this article are solely those of the authors and do not necessarily represent those of their affiliated organizations, or those of the publisher, the editors and the reviewers. Any product that may be evaluated in this article, or claim that may be made by its manufacturer, is not guaranteed or endorsed by the publisher.

## Footnotes

1
https://surfer.nmr.mgh.harvard.edu

2
http://enigma.ini.usc.edu/enigma-vis

3
https://www.proteinatlas.org/ENSG00000132639-SNAP25/tissue#top

## Supplementary Material

Supplementary Figure 1 Association Heatmaps between *SNAP-25* genotypes and morphological brain features. Dark areas represents significant associations according to the Benjamini–Hochberg procedure. “All statistical significances are in the range between 0.05 and 0.01.”

Supplementary Figure 2 Association Heatmaps between WISC-III scores and morphological brain features. Dark areas represents significant associations according to the Benjamini–Hochberg procedure. “All statistical significances are in the range between 0.05 and 0.01.”
