# Supplementary figures and images for: SNAP-25 Single Nucleotide Polymorphisms, Brain Morphology and Intelligence in Children With Borderline Intellectual Functioning: A Mediation Analysis
Source: Front Neurosci. 2021 Aug 26;15:715048. doi: 10.3389/fnins.2021.715048 (PMC8427043; doi:10.3389/fnins.2021.715048)

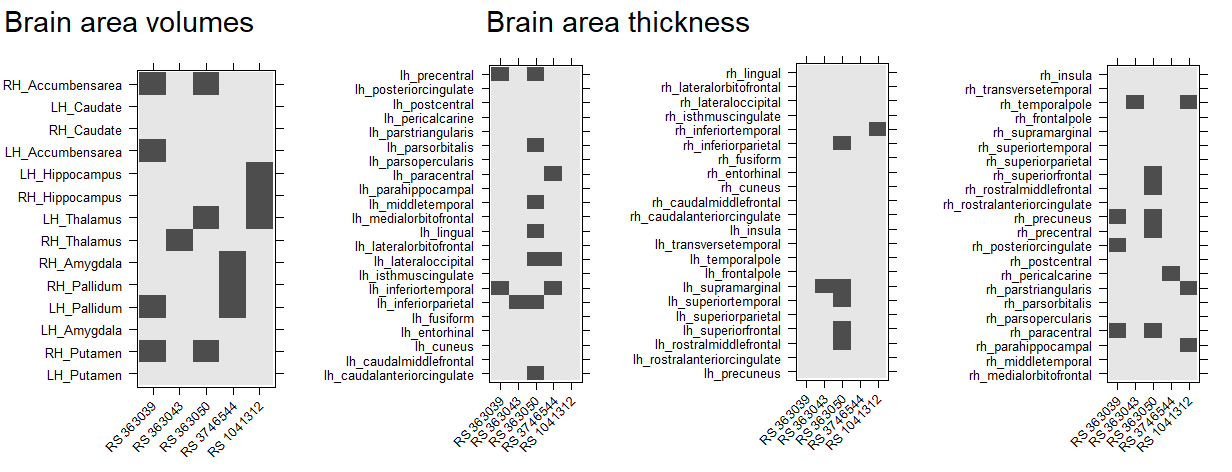

Supplement: Supplementary Figure 1 — Association Heatmaps between SNAP-25 genotypes and morphological brain features. Dark areas represents significant associations according to the Benjamini–Hochberg procedure. “All statistical significances are in the range between 0.05 and 0.01.” [file Image_1.TIF]

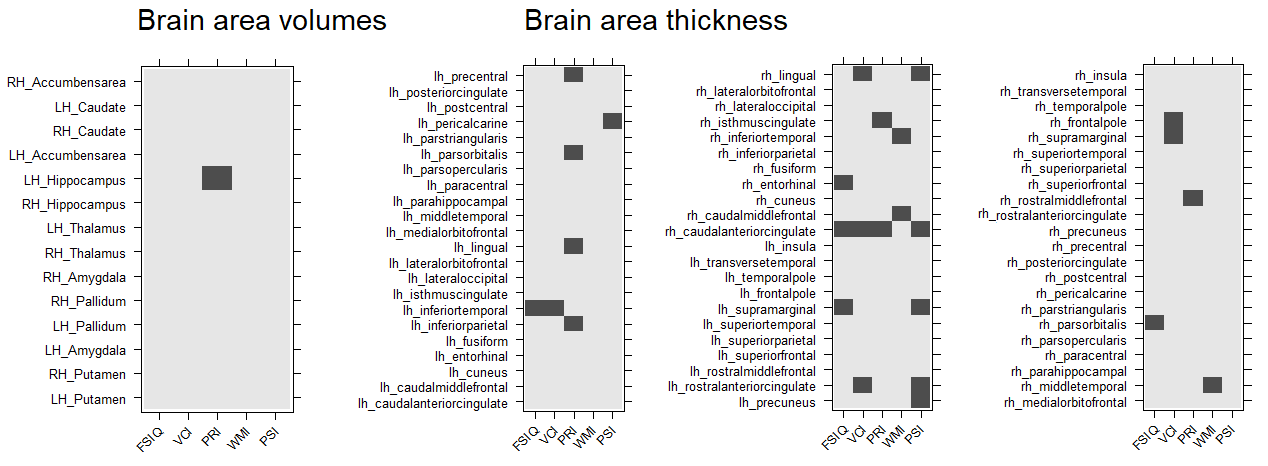

Supplement: Supplementary Figure 2 — Association Heatmaps between WISC-III scores and morphological brain features. Dark areas represents significant associations according to the Benjamini–Hochberg procedure. “All statistical significances are in the range between 0.05 and 0.01.” [file Image_2.TIF]
